# Heat Shock Protein 27 Plays a Pivotal Role in Myofibroblast Differentiation and in the Development of Bleomycin-Induced Pulmonary Fibrosis

**DOI:** 10.1371/journal.pone.0148998

**Published:** 2016-02-09

**Authors:** Ah-Mee Park, Kyosuke Kanai, Tatsuki Itoh, Takao Sato, Tatsuya Tsukui, Yutaka Inagaki, Moises Selman, Kouji Matsushima, Osamu Yoshie

**Affiliations:** 1 Department of Microbiology and Kindai University Faculty of Medicine, Osaka-Sayama, Osaka, Japan; 2 Department of Pathology, Kindai University Faculty of Medicine, Osaka-Sayama, Osaka, Japan; 3 Department of Molecular Preventive Medicine, Graduate School of Medicine, University of Tokyo, Tokyo, Japan; 4 Department of Regenerative Medicine, Tokai University School of Medicine, Sagamihara, Kanagawa, Japan; 5 Instituto Nacional de Enfermedades Respiratorias, México DF, Mexico; Medical University of South Carolina, UNITED STATES

## Abstract

Heat shock protein 27 (HSP27) is a member of the small molecular weight HSP family. Upon treatment with transforming growth factor β1 (TGF-β1), we observed upregulation of HSP27 along with that of α-smooth muscle actin (α-SMA), a marker of myofibroblast differentiation, in cultured human and mouse lung fibroblasts. Furthermore, by using siRNA knockdown, we demonstrated that HSP27 was involved in cell survival and upregulation of fibronectin, osteopontin (OPN) and type 1 collagen, all functional markers of myofibroblast differentiation, in TGF-β1-treated MRC-5 cells. In lung tissues of bleomycin-treated mice, HSP27 was strongly upregulated and substantially co-localized with α-SMA, OPN and type I collagen but not with proSP-C (a marker of type II alveolar epithelial cells), E-cadherin (a marker of epithelial cells) or F4/80 (a marker of macrophages). A similar co-localization of HSP27 and α-SMA was observed in lung tissues of patients with idiopathic pulmonary fibrosis. Furthermore, airway delivery of HSP27 siRNA effectively suppressed bleomycin-induced pulmonary fibrosis in mice. Collectively, our findings indicate that HSP27 is critically involved in myofibroblast differentiation of lung fibroblasts and may be a promising therapeutic target for lung fibrotic diseases.

## Introduction

Fibrosis is the end stage of persistent tissue damage and chronic inflammatory reactions. It is characterized by the excessive accumulation of extracellular matrix (ECM) and disruption of normal tissue architecture [1, 2]. Myofibroblasts are the major effector cells in tissue repair and fibrosis through their copious production of ECM proteins [3, 4]. Myofibroblasts differ from conventional fibroblasts by the elevated expression of α-smooth muscle actin (α-SMA) [3, 4], which contributes to enhanced motility and contractibility. Myofibroblasts are considered to be generated from a variety of cellular sources such as tissue resident mesenchymal cells including pericytes, bone marrow-derived progenitor cells (fibrocytes), and even epithelial/endothelial cells through the process of epithelial/endothelial-mesenchymal transition (EMT/EndoMT) [3, 5]. Transforming growth factor β1 (TGF-β1), which plays a key role in tissue repair and fibrosis, is also a potent inducer of myofibroblast differentiation [3, 5].

HSP27, also known as HSPB1, is a member of the small molecular weight HSP family that shares the conserved C-terminal α-crystallin domain [6, 7]. In response to heat shock, HSP27 functions as a protein chaperone to facilitate the proper refolding of damaged proteins. Furthermore, HSP27 is involved in a wide range of cellular processes such as survival, growth, differentiation, redox metabolism, and even tumorigenesis [6, 8]. Biological functions of HSP27 are also regulated through phosphorylation of its serine residues [6]. For example, HSP27 in its non-phosphorylated state prevents actin polymerization by functioning as an actin capping protein and thus inhibiting the fixation of a new actin monomer to the plus end of actin filament. On the other hand, phosphorylated HSP27 prevents actin filament degradation and promotes actin polymerization [7]. Previous proteomic studies revealed upregulation of HSP27 in human lung fibroblast cell lines HFL-1 and MRC-5 upon treatment with TGF-ß1 [4, 9, 10]. A proteomic study comparing lung tissues from IPF patients and control donors also demonstrated increases in HSP27 in IPF lung tissues [11]. However, the role of HSP27 in myofibroblast differentiation and function remains mostly unknown.

In the present study, we have corroborated upregulation of HSP27 at both mRNA and protein levels in TGF-ß1-treated human and mouse lung fibroblasts. We have also shown that HSP27 is critically involved in TGF-β1-induced upregulation of myofibroblast markers such as fibronectin (FN), type I collagen and osteopontin (OPN). We have further shown that HSP27 is strongly upregulated and su bstantially colocalized with the myofibroblast markers in lung tissues from bleomycin-treated mice and from patients with idiopathic pulmonary fibrosis (IPF). Finally, we have demonstrated that intranasal delivery of HSP27 siRNA effectively attenuates bleomycin-induced pulmonary fibrosis (PF) in mice. Collectively, upregulation of HSP27 plays a pivotal role in myofibroblast differentiation and may be a promising therapeutic target for fibrotic diseases including IPF.

## Materials and Methods

### Reagents

Recombinant TGF-β1 was purchased from R&D Systems (Minneapolis, MN). Mouse monoclonal antibodies against human α-SMA, α-tubulin, and β-actin and a rabbit antibody to FN were purchased from Sigma-Aldrich (St Louis, MO). A rabbit antibody to mouse α-SMA and a rat antibody to F4/80 were purchased from Abcam (Cambridge, MA) and AbD Serotec (Killington, UK), respectively. A rabbit antibody to prosurfactant proteins-C (proSP-C) and a rabbit antibody to OPN were purchased from Millipore (Temecula, CA) and Rockland (Limeruck, PA), respectively. A rabbit antibody to HSP27 and a goat antibody to HSP27 were purchased from Signalway Antibody (Pearland, TX) and Santa Cruz Biotechnology (Santa Cruz, CA), respectively. A rabbit antibody to phospho-HSP27 (Ser-82) was purchased from Cell Signaling (Beverly, MA). Secondary antibodies conjugated with horseradish peroxidase (HRP) were purchased from GE Healthcare (Buckinghamshire, UK). Bleomycin sulfate was purchased from Wako Pure Chemicals (Osaka, Japan).

### Cell Culture

MRC-5, a human lung fibroblast cell line, was obtained from Riken Cell Bank (Tsukuba, Japan) and cultured in Dulbecco’s modified Eagle’s medium (DMEM) supplemented with 10% fetal bovine serum (FBS) and antibiotics at 37°C in 5% CO_2_/95% air. Normal human lung fibroblasts (NHLF) were purchased from Lonza Japan (Tokyo, Japan) and cultured in Fibroblast Media containing human recombinant fibroblast growth factor β, insulin, FBS, and gentamycin/amphotenricin B. Normal mouse lung fibroblasts (NMLF) were prepared from CD1 mice as described previously [12] and cultured in DMEM supplemented with 10% FBS and antibiotics. Cells were washed twice with phosphate buffered saline (PBS) to remove FBS, cultured in serum-free Opti-MEM (Gibco) for 24 h, and treated with TGF-β1 to induce myofibroblast differentiation.

### Mice

CD1 mice were obtained from Charles River Laboratories Japan (Yokohama, Japan). Col1a2-GFP mice were described previously [13, 14]. Mice were housed in specific pathogen-free facilities and used for experiments at 8 to 10 weeks of age. The animal experiments were approved by the Institutional Animal Care and Use Committee of Kindai University (the authorization number: KAME-26-035) and performed in accordance with the institutional guidelines.

### Immunoblot Analysis

Cells were washed with PBS and lysed in CelLytic M (Sigma-Aldrich) containing Protease Inhibitor Cocktail Complete (Roche Diagnostics, Mannheim, Germany) and Phosphatase Inhibitor Cocktail (Toyobo, Osaka, Japan). After 15 min incubation at room temperature, cell debris was removed by centrifugation. Cell lysates were electrophoresed on a sodium dodecyl sulfate (SDS)-polyacrylamide gel in reducing conditions and electrophoretically transferred to a PVDF membrane. The membranes were blocked in 5% skim milk and probed with primary antibodies. After washing, the membranes were incubated with HRP-conjugated secondary antibodies and developed using ECL Prime System (GE Healthcare). Signal intensities were quantified using Image J software (NIH, Bethesda, MD).

### Quantitative PCR

Total RNAs were extracted from cells using RNeasy mini kit (Qiagen). RNA samples (500 ng each) were reverse-transcribed using High capacity RNA-to-cDNA kit (Applied Biosystems, Foster City, CA). Quantitative PCR (qPCR) was performed using Power SYBR Green PCR Master Mix (Applied Biosystems) and ABI Step One Real-Time PCR System, with glyceraldehyde-3-phosphate dehydrogenase (GAPDH) as a reference control. The human sequences of the sense and antisense primers were as follows: α-SMA, 5'-TGAGCGTGGCTATTCCTTCGT-3' and 5'-GCAGTGGCCATCTCATTTTCAA-3'; HSP27, 5'-TCCCTGGATGTCAACCACTTC-3' and 5'-TCTCCACCACGCCATCCT-3'; FN1, 5'-AGCCTGGGAGCTCTATTCCA-3' and 5'-CTTGGTCGTACACCCAGCTT-3'; COL1A1, 5'-CGAAGACATCCCACCAATCAC-3' and 5'-ACAGATCACGTCATCGCACAA-3'; OPN, 5'-GCATCACCTGTGCCATACCA-3' and 5'-GTGGGGCTAGGAGATTCTGC-3'; GAPDH, 5'-GATTCCACCCATGGCAAATT-3' and 5'-GATGGTGATGGGATTTCCATTG-3'. The mouse sequences of the sense and antisense primers were: α-SMA, 5'-TGAGCGTGGCTATTCCTTCGT-3' and 5'-GCCGTGGCCATCTCATTTTCAA-3'; HSP27, 5'-CCCTGGACGTCAACCACTTC-3' and 5'-ACGCCTTCCTTGGTCTTCACT-3'; GAPDH, 5'-CCTGCACCACCAACTGCTTAG-3' and 5'-GTGGATGCAGGGATGATGTTC-3'.

### In Vitro siRNA Transfection

HSP27 siRNA duplex (sense, 5' -UGAGAGACUGCCGCCAAGUAA-3'; antisense, 5'-UUACUUGGCGGCAGUCUC AUU-3') was obtained from Gene Design (Osaka, Japan). Non-targeting control siRNA was obtained from Cell Signaling. Transfection of siRNA was performed using Lipofectamine RNAiMax (Invitrogen). In brief, MRC-5 cells were cultured in multi-well plates at 4×10^4^ cells/ml in Opti-MEM containing 5% FBS. After 24 h, cells were transfected with 20 nM of control or HSP27 siRNA. After 24 h, cells were washed to remove FBS and further cultured in Opti-MEM without or with 0.5 ng/ml of TGF-β1. In some experiments, 2% FBS was added to Opti-MEM to reduce cellular damages caused by HSP27 knockdown. For quantitative PCR and immunoblot analysis, samples were obtained at 24 h and 48 h after TGF-β1 treatment, respectively.

### Evaluation of Cell Viability

Dead cells were stained *in situ* with propidium iodide (PI) at 5 μg/ml. PI-positive cells were counted on a fluorescent microscope. Cell apoptosis was evaluated by flow cytometric analysis. Briefly, cells were harvested by trypsinization and resuspended in a binding buffer (Immuno-Biological Laboratory, Gunma, Japan) containing Alexa Flour 647-Annexin V (Biolegend, San Diego, CA.) and 7-Amino-Actinomycin D (7AAD) (BD Biosciences, San Jose, CA.). After 15 min at room temperature in the dark, a minimum of 10,000 cells were immediately counted on a flow cytometer (LSRFortessa X-20, BD). Data were analyzed with the FlowJo software.

### Bleomycin-induced Pulmonary Fibrosis

Bleomycin sulfate was dissolved in PBS and intratracheally instilled at a dose of 1.5 mg/kg body weight. On day 14, mice were sacrificed and lungs were removed. Left lungs were fixed in 10% formaldehyde, embedded in paraffin, and sectioned. Tissue sections of 4 μm thickness were stained with hematoxlin-eosin (HE) and Masson's Trichrome. The severity of lung fibrosis was determined using a semiquantitative histopathological scoring method [15]. In brief, upon 100× magnification, each randomly selected field was scored ranging from 0 (normal lung) to 8 (severe distortion of structure, large fibrous areas, and honeycomb lesions). The scores from five fields were averaged for each sample. Immunohistochemistry was performed by the standard procedure using anti-HSP27 antibody as primary antibody and Histofine SAB-PO kit (Nichirei Biosciences; Tokyo, Japan). Immunofluorescence staining was also performed by the standard procedure using primary antibodies to HSP27, α-SMA, OPN, E-cadherin, proSP-C, and F4/80, and secondary antibodies labeled with Alexa Fluor 488 or Alexa Fluor 555 (Life Technologies, Carlsbad, CA.). For nuclear staining, TO-PRO-3 (Life Technologies) was used. Images were taken on a confocal laser microscope. Right lungs were homogenized in water. Hydroxyproline contents were determined as described previously [16]. For immunoblot analysis, lung homogenates were mixed with the same volume of 2× lysis buffer containing Complete Protease Inhibitor Cocktail (Santa Cruz Biotechnology) to make 10 mM HEPES pH 7.5, 2 mM EDTA, 2 mM EGTA, 1 mM NaF, 0.1 mM Na_3_VO_4_, 1 mM PMSF, and 1% Triton X-100. Immunoblot analysis was performed as described above.

### Human Studies

Formalin-fixed and paraffin-embedded human lung tissues were obtained from the archival tissue collections in the Department of Pathology, Kindai University Faculty of Medicine. For immunohistochemistry, lung tissue sections were stained using anti-HSP27 antibody and Histofine SAB-PO kit (Nichirei Biosciences; Tokyo, Japan). For immunofluorescence staining, lung tissue sections were reacted with primary antibodies to HSP27 and α-SMA, and then with secondary antibodies labeled with Alexa Fluor 488 or Alexa Fluor 555. Images were taken on a confocal laser microscope. Bronchoalveolar lavage (BAL) samples were obtained from 6 patients with IPF (5 males and 1 female, 67 ± 5.9 years) and 3 healthy individuals (3 males; 38 ± 7.6 years) as described previously [17]. Diagnosis was performed according to the American Thoracic Society/European Respiratory Society consensus [18]. None of the patients had been treated with corticosteroids or immunosuppressive drugs at the time of study. HSP27 contents in BAL samples were measured by using Human Total HSP27 DuoSet IC ELISA (R&D Systems). This study was approved by the Institutional Review Board of Kindai University Faculty of Medicine (the authorization number: 24–191) and by the Institutional Review Boards (Ethics Committee in Research and Research Committee) of Instituto Nacional de Enfermendades Respiratorias (the authorization number C54A-13). A written informed consent was obtained from all participants.

### In Vivo Delivery of siRNA

HSP27 siRNA (AAGGCGTGGTGGAGATCACTG) and control siRNA (TAAGGCTATGAAGAGATAC) were obtained from Gene Design (Osaka, Japan). Mice were intranasally inoculated with 20 μl MAX Suppressor In Vivo RNA-LANCEr II (Bioo Scientific, Austin, TX) containing 5 μg siRNA on day 4, 6, 9, 12 after bleomycin instillation. The efficiency of *in vivo* delivery of siRNA in the lung tissues was examined as follows. Four days after bleomycin instillation, mice were intranasally inoculated with 20 μl MAX Suppressor In Vivo RNA-LANCEr II containing 5 μg FITC-labeled control siRNA (Bioneer Corporation, Daejeon, Republic of Korea). One hour after inoculation, lungs were removed, embedded in OCT compound and frozen. Thin sections were made and fixed with 4% paraformaldehyde. Immunofluorescent images were taken on a confocal laser microscope.

### Statistical Analysis

Quantitative data are presented as the mean ± SE. Statistical analysis was performed using Student's *t*-test or one-way ANOVA. A *p* value of <0.05 was considered statistically significant.

## Results

### TGF-β1 Upregulates HSP27 Expression in Lung Fibroblasts

**Fig 1:**
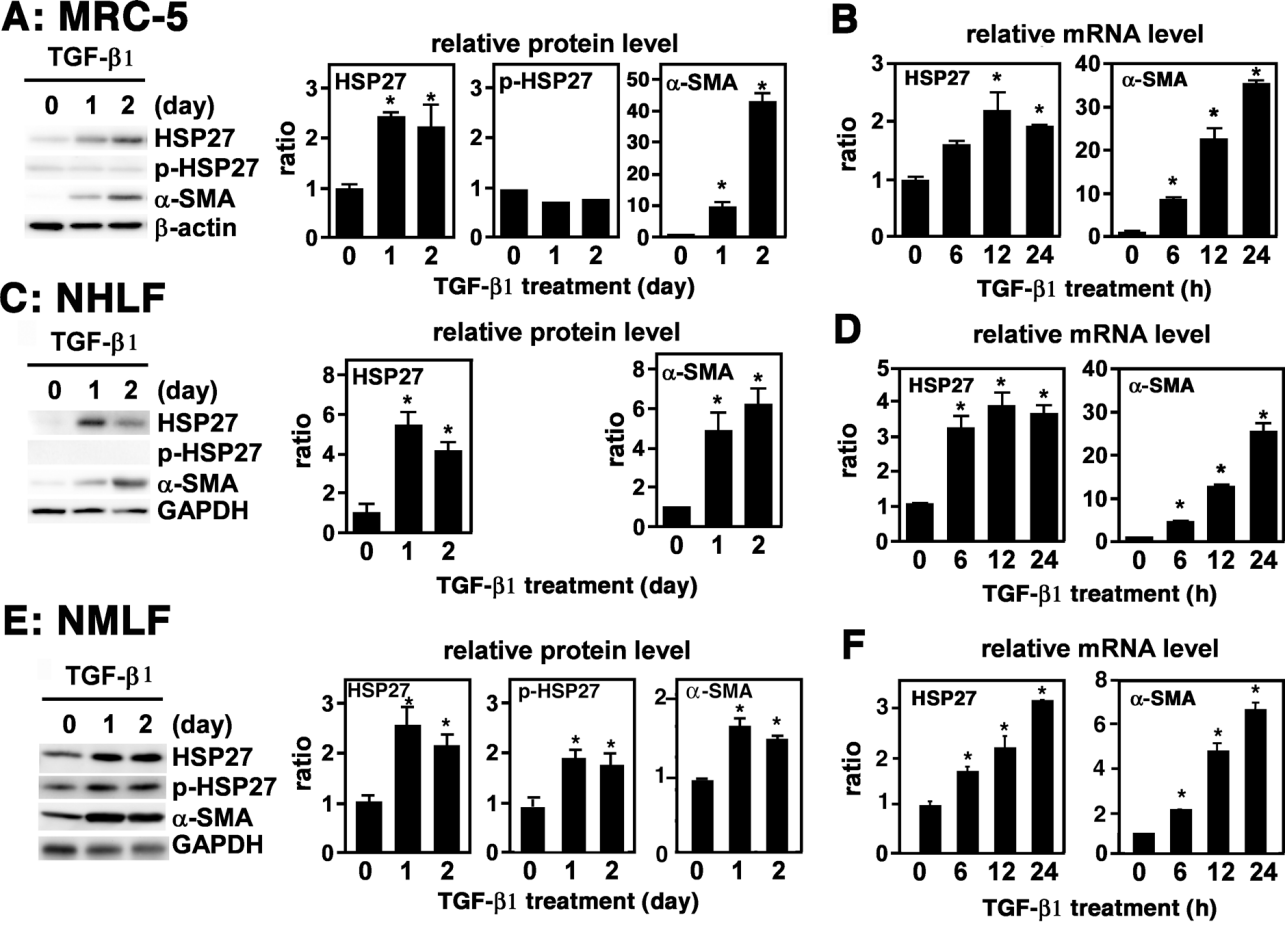
Upregulation of HSP27 in TGF-β1-treated lung fibroblasts. (A, B) MRC-5. Cells in monolayer were washed twice with PBS to remove FBS, cultured in Opti-MEM for 24 h, and mock-treated or treated with 1 ng/ml of TGF-β1 for the indicated lengths of time. (A) Immunoblot analysis. Protein levels of HSP27, p-HSP27, and α-SMA were determined by immunoblot analysis. For a loading control, β-actin was used. Signal intensities were quantified using Image J software and normalized by β-actin. A representative image from four independent experiments is shown in the left. Quantitative data are shown as mean ± SE (n = 4) in the right. *: P<0.05 by one-way ANOVA. (B) Quantitative PCR. Expression levels of HSP27 and α-SMA mRNAs were determined by quantitative PCR and normalized by GAPDH. Data are shown as mean ± SE (n = 4). *: P<0.05 by one-way ANOVA. (C, D) NHLF. Cells in monolayer were washed twice with PBS, cultured in Opti-MEM for 24 h, and mock-treated or treated with 2 ng/ml of TGF-β1 for indicated length of time. (C) Immunoblot analysis. This was performed as described above. For a loading control, GAPDH was used. A representative image from four independent experiments is shown in the left. Quantitative data are shown as mean ± SE (n = 4) in the right. *: P<0.05 by one-way ANOVA. (D) Quantitative PCR. This was performed as described above. Data are shown as mean ± SE (n = 4). *: P<0.05 by one-way ANOVA. (E, F) NMLF. Cells in monolayer were washed twice with PBS, cultured in Opti-MEM containing 1% FBS for 24 h, and mock-treated or treated with 4 ng/ml of TGF-β1 for the indicated length of time. (E) Immunoblot analysis. This was performed as described above. For a loading control, GAPDH was used. A representative image from six independent experiments is shown in the left. Quantitative data are shown as mean ± SE (n = 6) in the right. *: P<0.05 by one-way ANOVA. (F) Quantitative PCR. This was performed as described above. Data are shown as mean ± SE (n = 6). *: P<0.05 by one-way ANOVA.

MRC-5 is a human lung fibroblastic cell line that is frequently used as an *in vitro* model of myofibroblast differentiation [19, 20]. TGF-β1 is known to induce phenotypic changes in MRC-5 cells that include spindle-shaped morphology and upregulation of α-SMA, fibronectin (FN) and type I collagen, all signs of myofibroblast differentiation [21]. A previous proteomic study listed HSP27 as one of the cellular proteins upregulated in MRC-5 cells upon treatment with TGF-β1 [4]. To corroborate and extend this finding, we cultured MRC-5 cells in Opti-MEM without FBS for 24 h and treated with 1 ng/ml of TGF-β1. Of note, since FBS alone substantially upregulates myofibroblast differentiation markers in MRC-5, so the treatment with TGF-β1 needs to be performed in serum-free conditions (data not shown). Immunoblot analysis showed strong increase in HSP27 as well as α-SMA in TGF-β1-treated MRC-5 cells (Fig 1A). HSP27 phosphorylation levels were not appreciably affected (Fig 1A). Quantitative PCR also showed marked increase of α-SMA mRNA and moderate increase of HSP27 mRNA in TGF-β1-treated MRC-5 cells (Fig 1B). Very similar results were obtained from primary cultures of normal human lung fibroblasts (NHLF) (Fig 1C and 1D), although the levels of phosphorylated HSP27 (p-HSP) were very low, and of normal mouse lung fibroblasts (NMLF) (Fig 1E and 1F). Of note, NMLF required 1% FBS in Opti-MEM before and during TGF-β1 treatment to maintain cell viability. Thus, the expression levels of HSP27, p-HSP27 and α-SMA in NMLF were substantially elevated even on day 0 due to the effect of 1% FBS (Fig 1E). Thus, TGF-β1 upregulates the expression of HSP27 without affecting its phosphorylation level in cultured human and mouse lung fibroblasts.

### Effect of HSP27 Knockdown on TGF-β1-treated MRC-5

**Fig 2:**
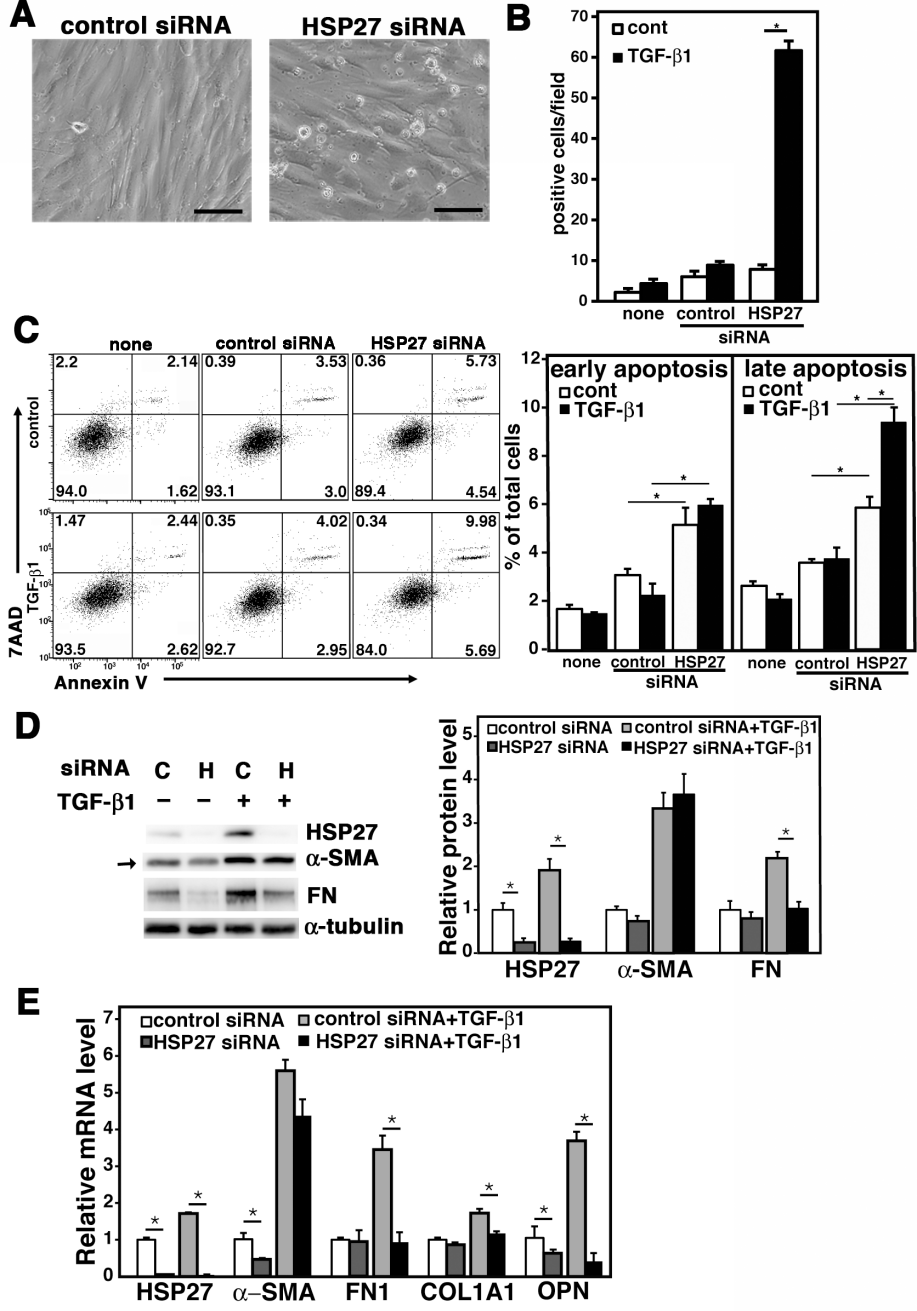
Effect of HSP27 siRNA on TGF-β1-treated MRC-5. MRC-5 cells were transfected with control or HSP27 siRNA and cultured for 24 h. After washing to remove FBS, cells were placed in Opti-MEM and treated with 0.5 ng/ml of TGF-β1 for 48 h. (A) Phase contrast microscopic images. Representative results from three independent experiments are shown. The bars indicate 100 μm. (B) Cell viability assay. Dead cells were detected by staining *in situ* with 5 μg/ml of propidium iotide (PI). PI-positive cells in each field (44 mm^2^) were counted on a fluorescence microscope. Data are shown as mean ± SE (n = 6). *: P<0.05 by Student’s *t*-test. (C) Apoptosis assay. Apoptotic cells were detected by flow cytometry using the Annexin V and 7AAD double staining assay. The FACS plots are shown in the left with % of cells in the four gated areas. Columns in the right show % of cells in early apoptosis (Annexin V^+^ and 7AAD^‒^) and late apoptotosis (Annexin V^+^ and 7AAD^+^) as mean ± SE (n = 3). *: P<0.05 by Student’s *t*-test. (D) Immunoblot assay. MRC-5 cells were transfected with control siRNA “C” or HSP27 siRNA “H” and cultured for 24 h. Then, after changing the culture medium to Opti-MEM containing 2% FBS, cells were treated with or without 0.5 ng/ml of TGF-β1 for 48 h. Protein levels of HSP27, α-SMA and fibronectin (FN) were determined by immunoblot analysis. For a loading control, α-tubulin was used. Quantitative data are shown as mean ± SE (n = 4) in the right. *: P<0.05 by Student’s *t*-test. (E) Quantitative PCR. MRC-5 cells were transfected with control or HSP27 siRNA and cultured for 24 h. Then, after changing the culture medium to Opti-MEM containing 2% FBS, cells were treated with or without 0.5 ng/ml of TGF-β1 for 24 h. Expression levels of HSP27, α-SMA, FN1, α1 type I collagen (COL1A1) and opsteopontin (OPN) mRNAs were determined by quantitative PCR and normalized by GAPDH. Data are shown as mean ± SE (n = 6). *: P<0.05 by Student’s *t*-test.

We next examined the role of HSP27 in TGF-β1-treated MRC-5 cells. To do this, MRC-5 cells were transfected with control or HSP27 siRNA. After 24 h, cells were washed to remove FBS and treated with 0.5 ng/ml of TGF-β1. As shown in Fig 2A, HSP27 siRNA caused strong cellular damage in TGF-β1-treated MRC-5 within 24 h. This led to a sharp increase in PI-positive dead cells *in situ* (Fig 2B). We also performed flow cytometric analysis to quantitate cells in early apoptosis (gated as Annexin V^+^ and 7AAD^‒^) and late apoptosis (gated as Annexin V^+^ and 7AAD^+^). The results also demonstrated that HSP27 siRNA significantly increased early apoptosis in both control and TGF-β1-treated MRC-5 cells and late apoptosis in TGF-β1-treated MRC-5 cells (Fig 2C). However, the values obtained from flow cytometric analysis could be quite underestimates because of the inevi loss of damaged cells during the preparation of suspension cells. Collectively, downregulation of HSP27 provoked cell death in TGF-β1-treated MRC-5 possibly due to damage in cell membrane integrity.

The above results prevented us from exploring the role of HSP27 in TGF-β1-treated MRC-5 cells under serum-free conditions. However, by adding 2% FBS during TGF-β1-treatment, we were able to protect cells from the cellular damage caused by HSP27 siRNA transfection. The problem was that 2% FBS itself already substantially upregulated α-SMA in MRC-5 cells even without TGF-β1-treatment (Fig 2D, arrow). Nevertheless, HSP27 siRNA decreased not only HSP27 itself but also FN, a marker of myofibroblast differentiation, at protein level in TGF-β1-treated MRC-5 cells (Fig 2D). Furthermore, by using quantitative PCR, we demonstrated that HSP27 siRNA significantly reduced the transcripts of not only HSP27 but also FN, α1 type I collagen (COL1A1), and OPN in TGF-β1-treated MRC-5 cells (Fig 2E). Of note, the gene encoding OPN (*Spp1*) was recently shown as one of the most highly upregulated genes in type I collagen-producing lung myofibroblasts during bleomycin-induced lung fibrosis [14]. Collectively, we concluded that HSP27 knockdown effectively inhibits myofibroblast differentiation of TGF-β1-treated MRC-5 cells, although it did not significantly affect the expression level of α-SMA, probably because of its strong upregulation by the combined effect of FBS and TGF-β1.

### Expression of HSP27 in Bleomycin-induced Pulmonary Fibrosis in Mice

**Fig 3:**
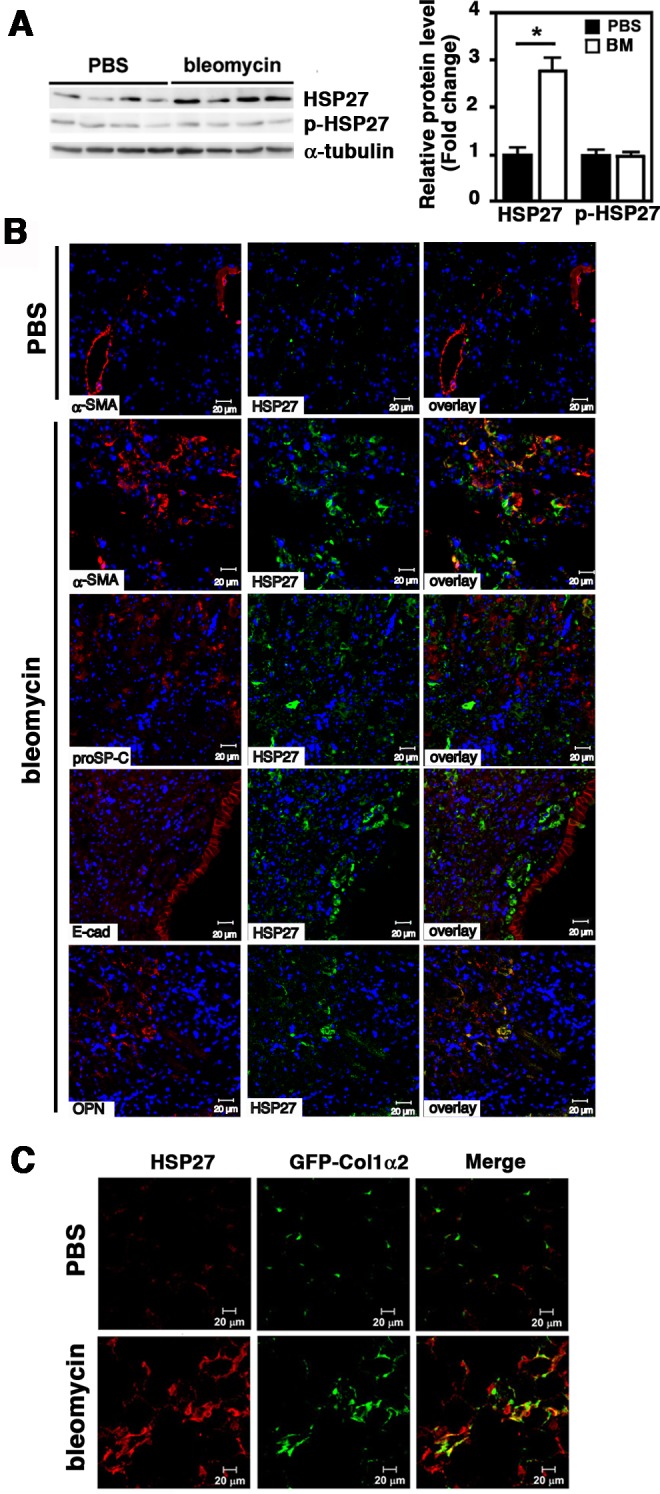
Upregulation of HSP27 in lung tissues of bleomycin-treated mice. Mice were intratracheally treated with PBS or bleomycin. After 14 days, mice were sacrificed and lungs were removed. (A) Immunoblot analysis. Protein levels of HSP27 and p-HSP27 were analyzed by Immunoblotting using tissue lysates prepared from right lungs. For a loading control, α-tubulin was used. Signal intensities were quantified using Image J software. A representative image from seven independent experiments is shown in the left. Quantitative data are shown as mean ± SE (n = 7) in the right. *: P<0.05 by Student’s *t*-test. (B) Immunofluorescence staining. Left lungs were fixed with 10% formaldehyde and embedded in paraffin. Tissue sections (4 μm) were double stained for HSP27 (green) and α-SMA (red), proSP-C (red), E-cadherin (E-cad, red) or OPN (red) as depicted. For nuclear staining, TO-PRO-3 (blue) was used. The bars indicate 20 μm. Representative images from three independent experiments are shown. (C) Col1a2-EGFP reporter mice. Mice were intratracheally instilled with PBS or bleomycin. After 14 days, mice were sacrificed and lungs were fixed with 4% paraformaldehyde, treated with 30% sucrose for cryoprotection, and embedded. Frozen sections (6 μm thick) were stained for HSP27 (red). Collagen Type I α2 was visualized by EGFP (green). The bars indicate 20 μm. Representative images from three independent experiments are shown.

We next employed bleomycin-induced pulmonary fibrosis in mice to examine HSP27 expression in lung myofibroblasts *in vivo*. Mice were intratracheally inoculated with PBS or bleomycin. After 14 days, mice were sacrificed and lungs were removed. By immunoblotting, HSP27 protein levels were clearly increased in bleomycin-treated lungs, although its phosphorylation level remained unchanged (Fig 3A). We then performed immunohistochemical staining of HSP27 along with various cellular markers (Fig 3B). In the lung tissues from PBS-treated mice, α-SMA staining was mostly confined to the airway and vascular smooth muscle cells, while HSP27 staining was only sparsely observed in the lung stroma (Fig 3B). In sharp contrast, there were dramatic increases in both α-SMA and HSP27 staining in bleomycin-treated lung tissues (Fig 3B). Furthermore, the signals of α-SMA and HSP27 substantially colocalized (Fig 3B). On the other hand, HSP27 did not colocalize with proSP-C (a marker of type II alveolar epithelial cells), E-cadherin (a marker of epithelial cells) (Fig 3B) or with F4/80 (a marker of macrophages; data not shown). Since the gene encoding OPN was found to be a downstream gene of HSP27 in TGF-β1-treated MRC-5 cells (Fig 2E), we also examined colocalization of OPN and HSP27. While OPN staining was mostly restricted to the airway epithelial cells in control lung tissues (data not shown), its signal was strongly upregulated in the stroma of bleomycin-treated lung tissues and mostly co-localized with HSP27 (Fig 3B). We further employed α2 type I collagen (Col1α2)-reporter mice (Col1a2-EGFP mice), in which collagen type I-positive cells express EGFP [13, 14]. In the lung tissues from PBS-treated Col1a2-EGFP mice, signals of HSP27 and GFP were only sparsely observed (Fig 3C). By contrast, HSP27 and GFP signals were remarkably increased in bleomycin-treated lung tissues (Fig 3C). Furthermore, HSP27 and GFP signals were substantially colocalized (Fig 3C). Collectively, these results clearly demonstrated strong upregulation of HSP27 in bleomycin-treated lung tissues and substantial colocalization of HSP27 with α-SMA, OPN, and type I collagen, all the markers of myofibroblasts. We therefore concluded that lung myofibroblasts strongly express HSP27 *in vivo*.

### Expression of HSP27 in Lung Tissues from IPF patients

**Fig 4:**
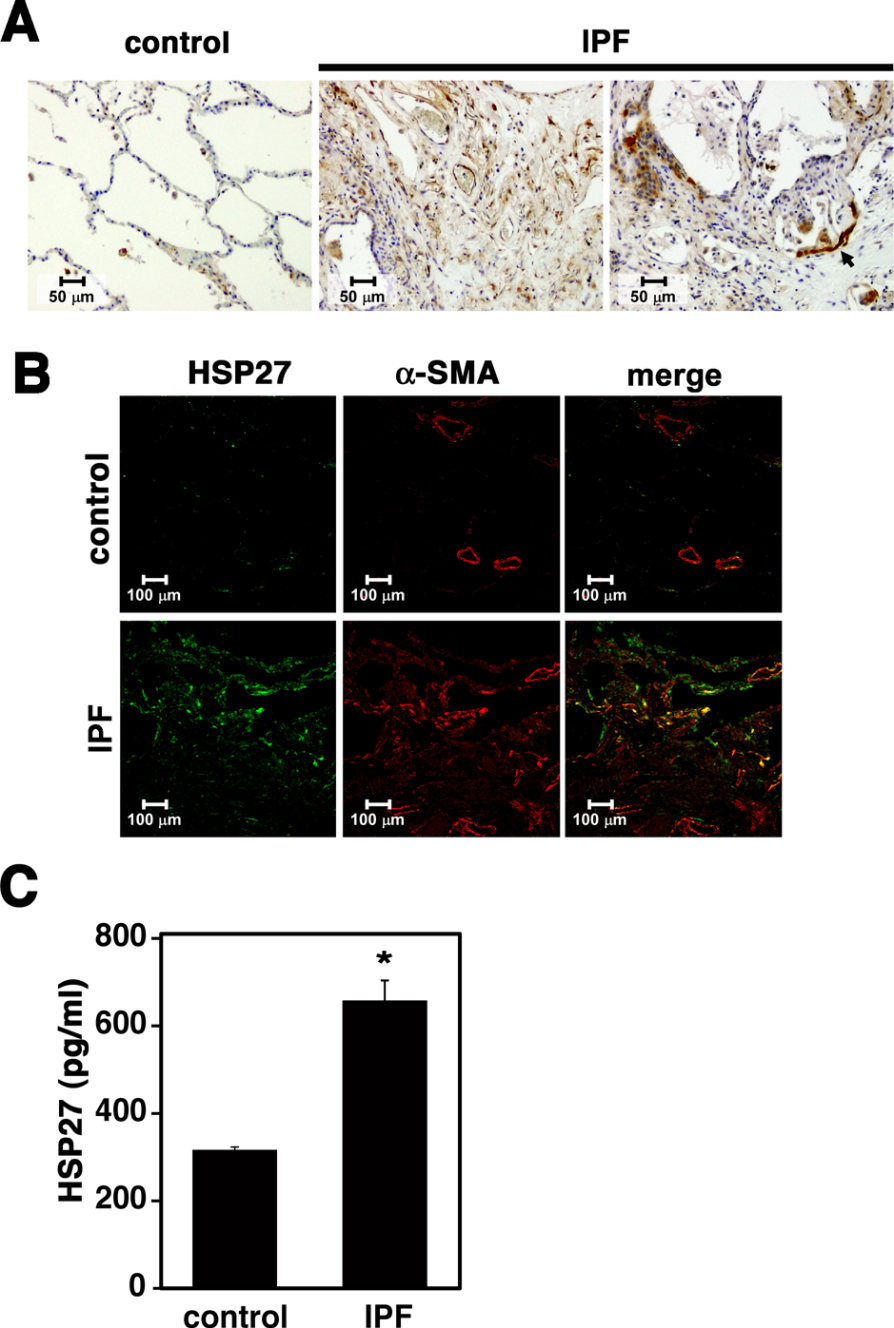
Strong upregulation of HSP27 in lung tissues from IPF patients. (A) Immunohistochemical staining of HSP27 in human lung tissues. Representative images are shown (n = 5). (B) Double immunofluorescence staining of HSP27 (green) and α-SMA (red) in human lung tissues. Representative images are shown (n = 4). (C) Quantitation of HSP27 in bronchoalveolar lavage (BAL) samples. HSP27 contents in BAL samples containing 0.5% Triton X-100 were determined by ELISA. Data are shown as mean ± SE (control, n = 3; IPF, n = 6). *: P<0.05 by Student’s *t*-test.

We next examined HSP27 expression in human lung tissues by immunohistochemistry (Fig 4A). While control lung tissues showed scarce HSP27 staining, strong HSP27 signals were seen in IPF lung tissues, especially in fibroblastic foci (FF). Furthermore, consistent with previous studies [11, 22], we observed strong HSP27 signals in bronchiolar epithelial cells adjacent to FF (arrows). We also performed double immunofluorescence staining of α-SMA and HSP27 (Fig 4B). In control lung tissues, α-SMA signals were mostly restricted to the airway and vessel smooth muscle cells, while HSP27 signals were sparsely observed. In IPF lung tissues, on the other hand, both α-SMA and HSP27 signals were strongly detected in FF and significantly overlapped. These results were quite consistent with those observed in lung tissues from bleomycin-treated mice (Fig 3). Furthermore, HSP27 contents in BAL fluids were significantly elevated in IPF patients (Fig 4C).

### Attenuation of Bleomycin-induced Pulmonary Fibrosis by HSP27 siRNA

**Fig 5:**
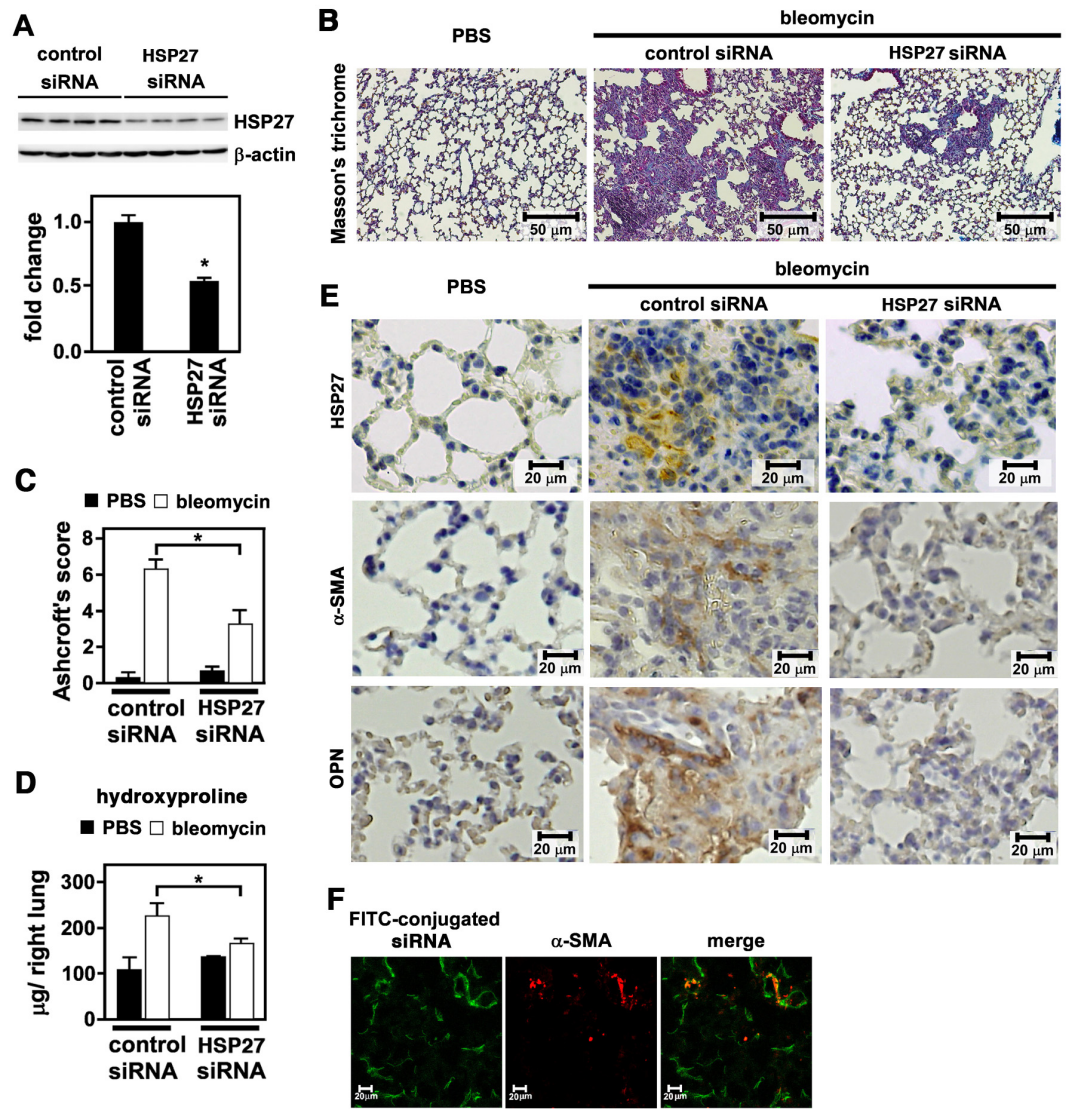
Effect of HSP27 siRNA on bleomycin-induced pulmonary fibrosis in mice. Mice were intratracheally treated with PBS or bleomycin. On day 4, 6, 9, and 12, mice were intranasally inoculated with 5 μg of control siRNA or HSP27 siRNA using MaxSuppressor In Vivo RNA-LANCEr II. On day 14, mice were sacrificed and lungs were removed. (A) Immunoblot analysis. Protein levels of HSP27 were analyzed by immunoblotting using tissue lysates prepared from right lungs. As a loading control, β-actin was used. A representative image from six independent experiments is shown (above). Quantitative data are shown as mean ± SE (n = 6) (below). *: P<0.05 by Student’s *t*-test. (B) Masson’s trichrome staining. This was performed using tissue sections from left lungs. Representative images from three independent experiments are shown. The bars indicate 50 μm. (C) Ashcroft’s Score. Fibrosis levels were quantified by Ashcroft’s Score. Data are shown as mean ± SE (n = 7). *: P<0.05 by Student’s *t*-test. (D) Hydroxyproline content. Tissue homogenates prepared from right lungs were used to determined hydroxproline contents. Data are shown as mean ± SE (n = 7). *: P<0.05 by Student’s *t*-test. (E) Immunohistochemical staining. Tissue sections from left lungs were immunohistochemically stained for HSP27, α-SMA, and OPN. Representative images from three independent experiments are shown. The bars indicate 20 μm. (F) Airway delivery of FITC-labeled siRNA. Mice were intratracheally treated with bleomycin. On day 4, mice were intranasally inoculated with 5 μg of FITC-labeled siRNA mixed in MaxSuppressor In Vivo RNA-LANCEr II. After 1 h, lungs were removed and frozen sections were made. Tissue sections were fixed and stained for α-SMA. FITC (green) and α-SMA (red) were observed on a fluorescence microscopy. Representative images from four independent experiments are shown.

The data so far indicate a critical role of HSP27 upregulation in lung myofibroblast differentiation and function. To prove this *in vivo*, we employed the siRNA-mediated approach to knock down HSP27 expression in the lungs of bleomycin-treated mice. After bleomycin inoculation, mice were intranasally delivered with control or HSP27 siRNA on day 4, 6, 9 and 12. On day 14, mice were sacrificed and lung tissues were examined. By immunoblotting of lung homogenates, we confirmed that HSP27 siRNA but not control siRNA significantly reduced HSP27 at protein levels (Fig 5A). Importantly, HSP27 siRNA but not control siRNA also effectively suppressed lung fibrosis as revealed by Masson’s Trichrome staining (Fig 5B), the Ashcroft’s score (Fig 5C), and hydroxyproline content (Fig 5D). By immunohistochemistry, we further confirmed reduction of HSP27, α-SMA and OPN in HSP27 siRNA-treated lung tissues compared to control siRNA-treated lung tissues (Fig 5E). Separately, we confirmed that FITC-labeled siRNA was efficiently distributed within lung tissues and incorporated into α-SMA-expressing cells (Fig 5F). Collectively, the airway delivery of HSP27 siRNA effectively suppressed bleomycin-induced lung fibrosis in mice.

## Discussion

Myofibroblasts play pivotal roles in tissue repair and fibrosis, and TGF-β1 is the major inducer of myofibroblast differentiation from various cell sources including fibroblasts [3, 5]. In the present study, we have shown that TGF-β1 strongly upregulates HSP27 along with α-SMA in cultured human and mouse lung fibroblasts. Furthermore, we have shown that the elevated expression of HSP27 is critically involved in cell survival and upregulation of several myofibroblast-associated proteins such as FN, type 1 collagen and OPN in TGF-β1-treated MRC-5. Of note, OPN is a matricellular protein and a proinflammatory cytokine that is known to be upregulated in various fibrotic diseases including IPF [23, 24]. In bleomycin-treated mouse lung tissues, OPN has been reported to be highly upregulated in type 1 collagen-expressing myofibroblasts [14]. Furthermore, the same study has shown that OPN expression and α-SMA expression represent different stages of myofibroblast differentiation, with OPN being the earlier marker [14]. Given that the kinetics of HSP27 upregulation was much faster than that of α-SMA upregulation in TGF-β1-treated MRC-5 and NHLF (Fig 1), HSP27 may also be an earlier marker of myofibroblast differentiation as OPN. We have further demonstrated strong upregulation of HSP27 and its colocalization with α-SMA, OPN, and type I collagen in lung tissues of bleomycin-treated mice (Fig 3). Similar results were obtained from lung tissues of IPF patients. Finally, we have shown effective attenuation of bleomycin-induced pulmonary fibrosis by airway delivery of HSP27 siRNA. Collectively, our findings indicate that upregulation of HSP27 is critically involved in myofibroblast differentiation from lung fibroblasts and HSP27 silencing has a strong therapeutic effect on bleomycin-induced lung fibrosis in mice.

Previous studies have also reported upregulation of HSP27 in lung tissues of patients with IPF/usual interstitial pneumonia [11, 22]. In these studies, HSP27 was found to be strongly associated with bronchiolar basal cells adjacent to fibroblastic foci (FF), the small collections of actively collagen-producing fibroblasts/myofibroblasts. Interestingly, HSP27-positive bronchiolar basal cells were arranged in a linear cluster located between the negative layer of overlying luminal epithelial cells and the underneath connective tissue, thus giving a sandwich appearance [11, 22]. It was speculated that the clusters of HSP27-positive bronchiolar basal cells were the sites of apoptosis and proliferation of type II alveolar epithelial cells undergoing cellular stress [11, 22]. However, the expression of HSP27 in lung myofibroblasts had not been closely examined. In the present study, we have clearly demonstrated the strong expression of HSP27 within FF and its substantial co-localization with α-SMA.

Previous studies demonstrated effective attenuation of bleomycin-induced lung fibrosis in rodents by airway delivery of siRNAs targeting IL-13 receptor α2 [25], NADPH oxidase-4 (NOX4) [26], plasminogen activator inhibitor-1 (PAI-1) [27], and the collagen-specific chaperon HSP47 [28]. Furthermore, Wettstein *et al*. recently showed that HSP27 siRNA effectively suppressed TGF-β1-induced subpleural fibrosis in rats [29]. In their experiments, subpleural lung fibrosis was induced by intrapleural injection of adenovirus expressing TGF-β1 (AdTGF-β1). Pleural mesothelial cells were transdifferentiated by locally produced TGF-β1 and acquired myofibroblast-like properties such as α-SMA expression and efficient migration into the pulmonary parenchyma [29]. Interestingly, they also observed strong upregulation of HSP27 in myofibroblasts originated from mesothelial cells [29]. Furthermore, they demonstrated using siRNA knockdown that HSP27 was crucial for TGF-β1-induced transdifferentiation of mesothelial cells to myofibroblasts *in vitro* [29]. They also showed that intrapeural injection of HSP27 siRNA effectively attenuated AdTGF-β1-induced subpleural fibrosis in rats [29]. Mechanistically, they further demonstrated that HSP27 promoted TGF-β1-induced transdifferentiation of mesothelial cells to myofibroblasts by protecting Snail from proteasomal degradation [29]. Snail is a known TGF-β1-inducible transcription repressor involved in EMT induction [30]. In the present study, however, we could not detect expression of Snail in lung fibroblasts (data not shown). Thus, Snail may not be involved in TGF-β1-induced myofibroblast differentiation of lung fibroblasts. The action mechanism of HSP27 in myofibroblast differentiation may thus differ considerably depending on the cell types. At any rate, the study by Wettstein *et al*. and our present findings clearly demonstrate the importance of HSP27 in TGF-β1-induced myofibroblast differentiation.

Upregulation of HSP27 has also been reported in kidney tubulointerstitial fibrosis in humans and in a rat experimental model [31, 32]. In the latter study, unilateral ureteral obstruction led to strong expression of TGF-β1 and HSP27 in tubular epithelial cells [32]. Furthermore, tubular epithelial cells were shown to undergo EMT as revealed by upregulation of α-SMA and downregulation of E-cadherin [32]. Surprisingly, however, overexpression of HSP27 in tubular epithelial cells *in vitro* increased the expression of E-cadherin and decreased Snail [33]. Furthermore, *in vivo* overexpression of HSP27 in tubular epithelial cells suppressed tubulointerstitial fibrosis [33]. Thus, in sharp contrast to the results reported by Wettstein *et al*. [29] and also shown in the present study, the role of HSP27 in TGF-β1-induced EMT of renal tubular epithelial cells appears to be opposite. The reason for such discrepancy is not clear at present but may be related to the difference in cell types.

The exact role of HSP27 in myofibroblast differentiation is not known at present, but may be related to its function in the regulation of actin filament network [7]. Thus, the upregulation of HSP27 may be closely associated with the locomotion and contractility of myofibroblasts. The strong cytotoxic effect of HSP27 siRNA on TGF-β1-treated MRC-5 may also indicate a crucial role of HSP27 in myofibroblast cell integrity. In fact, this cytotoxic effect could be in part the mechanism of the therapeutic effect of HSP27 siRNA on bleomycin-induced pulmonary fibrosis. However, we rather consider that the suppression of myofibroblast differentiation by HSP27 siRNA is the primary mechanism of its therapeutic effect on bleomycin-induced pulmonary fibrosis in mice. Currently, we are trying to identify HSP27-interacting proteins that are involved in TGF-β1-induced myofibroblast differentiation of lung fibroblasts.

HSP27 is also known to be overexpressed in various cancers and knockdown of HSP27 in cancer models induces cell death and tumor regression [34]. Accordingly, OGX-427, an HSP27-specific antisense oligonucleotide, is currently in clinical trials for various cancers [34]. The findings by Wettstein *et al*. [29] and the present study may warrant that HSP27 is also a potential therapeutic target for fibrotic diseases such as IPF.

## References

[1] Wight TN, Potter-Perigo S. The extracellular matrix: an active or passive player in fibrosis? Am J Physiol Gastrointest Liver Physiol. 2011; 301(6):G950–955. 10.1152/ajpgi.00132.2011 . 21512158 PMC3233785

[2] Selman M, King TE, Pardo A. Idiopathic pulmonary fibrosis: prevailing and evolving hypotheses about its pathogenesis and implications for therapy. Ann Intern Med. 2001; 134(2):136–151. . 11177318 10.7326/0003-4819-134-2-200101160-00015

[3] Wynn TA. Cellular and molecular mechanisms of fibrosis. J Pathol. 2008; 214(2):199–210. 10.1002/path.2277 . 18161745 PMC2693329

[4] Honda E, Park AM, Yoshida K, Tabuchi M, Munakata H. Myofibroblasts: Biochemical and proteomic approaches to fibrosis. Tohoku J Exp Med. 2013; 230(2):67–73. . 23774326 10.1620/tjem.230.67

[5] Willis BC, Borok Z. TGF-β-induced EMT: mechanisms and implications for fibrotic lung disease. Am J Physiol Lung Cell Mol Physiol. 2007; 293(3):L525–534. 10.1152/ajplung.00163.2007 . 17631612

[6] Kostenko S, Moens U. Heat shock protein 27 phosphorylation: kinases, phosphatases, functions and pathology. Cell Mol Life Sci. 2009; 66(20):3289–3307. 10.1007/s00018-009-0086-3 . 19593530 PMC11115724

[7] Wettstein G, Bellaye PS, Micheau O, Bonniaud P. Small heat shock proteins and the cytoskeleton: an essential interplay for cell integrity? Int J Biochem Cell Biol. 2012; 44(10):1680–1686. 10.1016/j.biocel.2012.05.024 . 22683760

[8] Christians ES, Ishiwata T, Benjamin IJ. Small heat shock proteins in redox metabolism: implications for cardiovascular diseases. Int J Biochem Cell Biol. 2012; 44(10):1632–1645. 10.1016/j.biocel.2012.06.006 . 22710345 PMC3412898

[9] Bratt C, Lindberg C, Marko-Varga G. Restricted access chromatographic sample preparation of low mass proteins expressed in human fibroblast cells for proteomics analysis. J Chromatogr A. 2001; 909(2):279–288. . 11269527 10.1016/s0021-9673(00)01103-1

[10] Malmstrom J, Lindberg H, Lindberg C, Bratt C, Wieslander E, Delander EL, et al Transforming growth factor-β1 specifically induce proteins involved in the myofibroblast contractile apparatus. Mol Cell Proteomics. 2004; 3(5):466–477. 10.1074/mcp.M300108-MCP200 . 14766930

[11] Korfei M, Schmitt S, Ruppert C, Henneke I, Markart P, Loeh B, et al Comparative proteomic analysis of lung tissue from patients with idiopathic pulmonary fibrosis (IPF) and lung transplant donor lungs. J. Proteome Res. 2011; 10(5):2185–2205. 10.1021/pr1009355 . 21319792

[12] Seluanov A, Vaidya A, Gorbunova V. Establishing primary adult fibroblast cultures from rodents. J Vis Exp. 2010; (44) 10.3791/2033 . 20972406 PMC3185624

[13] Higashiyama R, Moro T, Nakao S, Mikami K, Fukumitsu H, Ueda Y, et al Negligible contribution of bone marrow-derived cells to collagen production during hepatic fibrogenesis in mice. Gastroenterol. 2009; 137(4):1459–1466. 10.1053/j.gastro.2009.07.006 . 19596008

[14] Tsukui T, Ueha S, Abe J, Hashimoto S, Shichino S, Shimaoka T, et al Qualitative rather than quantitative changes are hallmarks of fibroblasts in bleomycin-induced pulmonary fibrosis. Am J Pathol. 2013; 183(3):758–773. 10.1016/j.ajpath.2013.06.005 . 23886891

[15] Ashcroft T, Simpson JM, Timbrell V. Simple method of estimating severity of pulmonary fibrosis on a numerical scale. J Clin Pathol. 1988; 41(4):467–470. . 3366935 10.1136/jcp.41.4.467 PMC1141479

[16] Thakur SA, Beamer CA, Migliaccio CT, Holian A. Critical role of MARCO in crystalline silica-induced pulmonary inflammation. Toxicol Sci. 2009; 108(2):462–471. 10.1093/toxsci/kfp011 . 19151164 PMC2664690

[17] Burkhardt AM, Tai KP, Flores-Guiterrez JP, Vilches-Cisneros N, Kamdar K, Barbosa-Quintana O, et al CXCL17 is a mucosal chemokine elevated in idiopathic pulmonary fibrosis that exhibits broad antimicrobial activity. J Immunol. 2012; 188(12):6399–6406. 10.4049/jimmunol.1102903 . 22611239 PMC3370106

[18] Raghu G, Collard HR, Egan JJ, Martinez FJ, Behr J, Brown KK, et al An official ATS/ERS/JRS/ALAT statement: idiopathic pulmonary fibrosis: evidence-based guidelines for diagnosis and management. Am J Respir Crit Care Med. 2011; 183(6):788–824. 10.1164/rccm.2009-040GL . 21471066 PMC5450933

[19] Yang Y, Fujita J, Bandoh S, Ohtsuki Y, Yamadori I, Yoshinouchi T, et al Detection of antivimentin antibody in sera of patients with idiopathic pulmonary fibrosis and non-specific interstitial pneumonia. Clin Exp Immunol. 2002; 128(1):169–174. . 11982605 10.1046/j.1365-2249.2002.01811.x PMC1906354

[20] Nakatani T, Honda E, Hayakawa S, Sato M, Satoh K, Kudo M, et al Effects of decorin on the expression of α-smooth muscle actin in a human myofibroblast cell line. Mol Cell Biochem. 2008; 308(1–2):201–207. 10.1007/s11010-007-9629-9 . 17952560

[21] Honda E, Yoshida K, Munakata H. Transforming growth factor-β upregulates the expression of integrin and related proteins in MRC-5 human myofibroblasts. Tohoku J Exp Med. 2010; 220(4):319–327. . 20410683 10.1620/tjem.220.319

[22] Chilosi M, Zamo A, Doglioni C, Reghellin D, Lestani M, Montagna L, et al Migratory marker expression in fibroblast foci of idiopathic pulmonary fibrosis. Respir Res. 2006; 7:95 10.1186/1465-9921-7-95 . 16813649 PMC1538593

[23] Pardo A, Gibson K, Cisneros J, Richards TJ, Yang Y, Becerril C, et al Up-regulation and profibrotic role of osteopontin in human idiopathic pulmonary fibrosis. PLoS Med. 2005; 2(9):e251 10.1371/journal.pmed.0020251 . 16128620 PMC1198037

[24] Foster MW, Morrison LD, Todd JL, Snyder LD, Thompson JW, Soderblom EJ, et al Quantitative proteomics of bronchoalveolar lavage fluid in idiopathic pulmonary fibrosis. J Proteome Res. 2015; 14(2):1238–1249. 10.1021/pr501149m . 25541672

[25] Fichtner-Feigl S, Strober W, Kawakami K, Puri RK, Kitani A. IL-13 signaling through the IL-13α2 receptor is involved in induction of TGF-β1 production and fibrosis. Nat Med. 2006; 12(1):99–106. 10.1038/nm1332 . 16327802

[26] Hecker L, Vittal R, Jones T, Jagirdar R, Luckhardt TR, Horowitz JC, et al NADPH oxidase-4 mediates myofibroblast activation and fibrogenic responses to lung injury. Nat Med. 2009; 15(9):1077–1081. 10.1038/nm.2005 . 19701206 PMC2743335

[27] Senoo T, Hattori N, Tanimoto T, Furonaka M, Ishikawa N, Fujitaka K, et al Suppression of plasminogen activator inhibitor-1 by RNA interference attenuates pulmonary fibrosis. Thorax. 2010; 65(4):334–340. 10.1136/thx.2009.119974 . 20388759

[28] Hagiwara S, Iwasaka H, Matsumoto S, Noguchi T. Antisense oligonucleotide inhibition of heat shock protein (HSP) 47 improves bleomycin-induced pulmonary fibrosis in rats. Respir Res. 2007; 8:37 10.1186/1465-9921-8-37 . 17504519 PMC1876458

[29] Wettstein G, Bellaye PS, Kolb M, Hammann A, Crestani B, Soler P, et al Inhibition of HSP27 blocks fibrosis development and EMT features by promoting Snail degradation. FASEB J. 2013; 27(4):1549–1560. 10.1096/fj.12-220053 . 23288928

[30] Zavadil J, Bottinger EP. TGF-β and epithelial-to-mesenchymal transitions. Oncogene. 2005; 24(37):5764–5774. 10.1038/sj.onc.1208927 . 16123809

[31] Valles P, Jorro F, Carrizo L, Manucha W, Oliva J, Cuello-Carrion FD, et al Heat shock proteins HSP27 and HSP70 in unilateral obstructed kidneys. Pediatric Nephrol. 2003; 18(6):527–535. 10.1007/s00467-003-1096-2 . 12698327

[32] Vidyasagar A, Reese S, Acun Z, Hullett D, Djamali A. HSP27 is involved in the pathogenesis of kidney tubulointerstitial fibrosis. Am J Physiol Renal Physiol. 2008; 295(3):F707–716. 10.1152/ajprenal.90240.2008 . 18596079 PMC2536879

[33] Vidyasagar A, Reese SR, Hafez O, Huang LJ, Swain WF, Jacobson LM, et al Tubular expression of heat-shock protein 27 inhibits fibrogenesis in obstructive nephropathy. Kidney Int. 2013; 83(1):84–92. 10.1038/ki.2012.336 . 22971995 PMC3525804

[34] Zoubeidi A, Gleave M. Small heat shock proteins in cancer therapy and prognosis. Int J Biochem Cell Biol. 2012; 44(10):1646–1656. 10.1016/j.biocel.2012.04.010 . 22571949

